# Bone Phenotype Assessed by HRpQCT and Associations with Fracture Risk in the GLOW Study

**DOI:** 10.1007/s00223-017-0325-9

**Published:** 2017-09-14

**Authors:** A. E. Litwic, L. D. Westbury, D. E. Robinson, K. A. Ward, C. Cooper, E. M. Dennison

**Affiliations:** 1 MRC Lifecourse Epidemiology Unit, University of Southampton, Southampton, UK; 2 Arthritis Research UK Centre for Epidemiology, Centre for Musculoskeletal Research, Manchester Academic Health Science Centre, The University of Manchester, Manchester, UK; 3 NIHR Musculoskeletal Biomedical Research Unit, University of Oxford, Oxford, UK

**Keywords:** HRpQCT, DXA, Osteoporosis, Epidemiology, Fracture risk assessment

## Abstract

The epidemiology and pathogenesis of fractures in postmenopausal women has previously been investigated in the Global Longitudinal study of Osteoporosis in Women (GLOW). To date, however, relationships between bone imaging outcomes and fracture have not been studied in this cohort. We examined relationships between high-resolution peripheral quantitative computed tomography (HRpQCT) parameters and fracture in the UK arm of GLOW, performing a cluster analysis to assess if our findings were similar to observations reported from older participants of the Hertfordshire Cohort Study (HCS), and extended the analysis to include tibial measurements. We recorded fracture events and performed HRpQCT of the distal radius and tibia and dual-energy X-ray absorptiometry (DXA) of the hip in 321 women, mean age 70.6 (SD 5.4) years, identifying four clusters at each site. We saw differing relationships at the radius and tibia. Two radial clusters (3 and 4) had a significantly lower hip areal bone mineral density (*p* < 0.001) compared to Cluster 1; only individuals in Cluster 4 had a significantly higher risk of fracture (*p* = 0.005). At the tibia, clusters 1, 3 and 4 had lower hip areal bone mineral density (*p* < 0.001) compared to Cluster 2; individuals in Cluster 3 had a significantly higher risk of fracture (*p* = 0.009). In GLOW our findings at the radius were very similar to those previously reported in the HCS, suggesting that combining variables derived from HRpQCT may give useful information regarding fracture risk in populations where this modality is available. Further data relating to tibial HRpQCT-phenotype and fractures are provided in this paper, and would benefit from validation in other studies. Differences observed may reflect age differences in the two cohorts.

## Introduction

Osteoporosis is a disease characterised by loss of bone mass and structural deterioration, resulting in increased bone fragility and propensity to fracture. It is a major public health problem, with a high impact on quality of life and high rates of morbidity. Worldwide, there are nearly nine million osteoporotic fractures each year [1]. The burden of fragility fractures will grow with ageing of the population; the US Surgeon General’s report of 2004, consistent with data from the UK, suggested that almost one in two women and one in five men will experience a fracture in their remaining lifetime from the age of 50 years [2]. The economic cost of osteoporosis and fractures are projected to increase in the EU from €37.4 billion in 2010 to €46.8 billion by 2025 and, in the US, from $17 billion in 2005 to $25.3 billion by 2025 [3, 4].

In clinical practice, the definition of osteoporosis relies on measurements of areal bone mineral density (aBMD) by dual-energy X-ray absorptiometry (DXA) [5]. While aBMD is a significant predictor of fracture risk, it is limited because of its two-dimensional nature, which is affected by the size and position of the subject and cannot distinguish between cortical and trabecular compartments. Epidemiological data indicate that a significant proportion of fractures occur in women who would not be classified as osteoporotic according to current aBMD criteria, highlighting the limitations of this approach and the need for other assessment methods to determine underlying causes of bone fragility [6, 7]. Recent advances in imaging permit the assessment of bone microstructure in vivo using high-resolution peripheral quantitative computed tomography (HRpQCT). This imaging modality has been utilized in research settings to examine factors, including skeletal properties of cortical bone and trabecular microarchitecture, that may contribute to fracture risk [8–12].

So far, most studies investigating aetiology of fracture have analysed specific components of bone structure assessing differences in single outcomes between fracture and non-fractured cases [8–11]. However, cluster analysis allows us to use the data derived from such scans to define bone phenotypes taking into account all parameters derived from HRpQCT scans. A recent study of older men and women, however, demonstrated that two separate phenotypes were associated with high fracture rates, using such mathematical cluster analysis of bone size, volumetric density (vBMD) and microarchitecture from HRpQCT [12]. In the first phenotype, cortical parameters differed with mean cortical thickness and cortical vBMD lower than the sample mean, whereas the second phenotype was characterised by deficiencies in predominantly trabecular bone with lower values than the sample mean. Replication of these findings in an unrelated cohort was a key conclusion of this study and was the rationale of undertaking this current work in the Global Longitudinal study of Osteoporosis in Women (GLOW) study. The epidemiology and pathogenesis of fractures in postmenopausal women has been widely investigated in GLOW—a prospective, multinational, observational, population-based study of postmenopausal women who were 55 years of age and older [13–20]. However, relationships between bone imaging outcomes and fracture rates have not previously been examined in this cohort. Women who participated in the UK component of the GLOW underwent DXA and HRpQCT of the distal radius and tibia. Extensive phenotyping of HRpQCT images allowed the assessment of relationships between individual HRpQCT parameters and fracture, and a cluster analysis which we undertook to assess if the findings were similar to observations reported in older participants of the Hertfordshire Cohort Study, and extended to the tibial site.

## Materials and Methods

### Study Participants

GLOW is a prospective, observational cohort study conducted through general physician practices in 10 countries. Study design and recruitment have been described in detail previously [21]. In brief, practices, representative of each region, were recruited through primary care networks and provided the names of women aged 55 years and older who had been seen by their physician in the past 24 months. The primary aim of GLOW was to characterise the descriptive epidemiology and health impact of osteoporosis-related fractures among women who were 55 years of age and older worldwide. Globally, GLOW enrolled over 60,000 women through over 700 physicians in 10 countries, and conducted annual follow-up for up to 5 years through annual patient questionnaires. In Southampton only, participants with baseline data and at least one follow-up questionnaire were invited, after completion of 5 years of follow-up, for a follow-up study which included DXA and HRpQCT. Participants were scanned between April 2014 and September 2016. Patients, who were institutionalized or were not able to complete the study survey by themselves due to cognitive impairment, language barriers, institutionalization, or were too ill to complete the survey or attend for the scans were excluded.

### Questionnaires

Information was collected using self-administered questionnaires and included details regarding smoking status, alcohol consumption, education level, use of anti-osteoporotic medication (AOM), years since menopause and use of oestrogen or hormone replacement therapy (HRT). Participants were also asked to rate how physically active they were compared to other women of the same age out of the following possible responses: ‘very active’, ‘somewhat active’, ‘a little’ and ‘not at all’. Subjects were considered to be taking anti-osteoporosis medication if, from baseline to the 5-year follow-up, they reported current use of alendronate, calcitonin, etidronate, ibandronate, pamidronate, raloxifene, risedronate, strontium ranelate, teriparatide, tibolone or zoledronic acid. Fracture history was ascertained at baseline and further information on fractures was obtained after 1-, 2-, 3- and 5-year follow-up. Fracture location included the following: clavicle, upper arm, wrist, spine, rib, hip, pelvis, ankle, upper leg and lower leg. Fractures that were reported at baseline, or accrued over 5 years of follow-up were included; hence the fractured subjects were those with prevalent fracture at the time of scan.

### Anthropometry and Dual-Energy X-ray Absorptiometry (DXA)

Height was measured to the nearest 0.1 cm using a Marsden stadiometer; weight was measured to the nearest 0.1 kg on the day of scanning using a Marsden MPPS-250 (Marsden Weighing Machine Group Limited, Rotherham, UK) digital floor scale.

Total hip areal bone mineral density (aBMD, g/cm^2^) was measured at both sides using DXA Hologic Horizon W; software version Apex 5.5.3.1 (Vertec Scientific, Reading, UK); the total effective dose equivalent of the hip scans was 4.7 microsieverts.

### Assessment of Bone by HRpQCT

Each participant underwent a HRpQCT scan of the non-dominant distal radius and tibia using XtremeCT I, (Scanco Medical, Basserdorf, Switzerland); if there was a history of fracture on the non-dominant limb, the non-fractured limb was measured. A stack of 110 parallel HRpQCT slices were acquired with an isotropic voxel size of 82 µm. Methods used to process the HRpQCT data have been described previously [9]. The standard evaluation and cortical porosity scripts were run to obtain estimates of total area, trabecular area, cortical area, cortical volumetric density, trabecular volumetric density, trabecular number, trabecular thickness, trabecular separation, cortical porosity and cortical thickness [22]. Of participants with radius scans, 93 of 442 participants had grade 5 scans and were excluded; of participants with tibial scans, 15 of 447 had grade 5 scans and were excluded. The main analysis sample consisted of 321 individuals with complete data on fracture history and the radial HRpQCT parameters; analysis of the tibial HRpQCT parameters was based on a subset of 306/321 participants who also had complete data on the tibial HRpQCT parameters.

### Statistical Analysis

Linear regression was used to examine the relationships between individual HRpQCT parameters and fracture history. Unadjusted and fully adjusted associations, accounting for age at time of HRpQCT scan, height, BMI, physical activity, smoking status, alcohol consumption, education, time since last period, use of AOM and oestrogen/HRT, were examined.

The k-means partitioning method of cluster analysis was used to produce clusters of the HRpQCT parameters for the tibia and radius separately. The number of clusters selected was based on the stability of the clustering, and on the potential for identifying contrasting phenotypes (12). The means and standard deviations (SD) of the standardized HRpQCT parameters, and fracture proportion were then determined for each cluster. Poisson regression with robust variance estimation was used to determine the likelihood of fracture in each cluster compared to the lowest risk cluster. Mean total hip aBMD in each cluster was compared to the cluster with the lowest fracture risk. Data were analysed using Stata, version 14.0.

## Results

The characteristics of the study population are shown in Table 1. The mean (SD) age of the 321 participants studied was 70.6 (5.4) years at the time of the radius scan. Overall, 63 (19.6%) women reported a fracture among at least one of the fracture locations. The most common fracture site was at the wrist with 25 fractures (32.5% of all fractures among the 10 fracture locations), followed by ankle [15 fractures], rib [11], lower leg [11], upper arm [6], spine [4], hip [2], clavicle [2], pelvis [1] and upper leg [0]. Less than 6% of women were smokers; and a vast majority (91%) did not exceed the recommended limits of alcohol intake.

**Table 1:** Participants’ characteristics

Participant characteristic	Mean (SD)
Age at baseline (years)	63.0 (5.4)
Age at radius scan (years)	70.6 (5.4)
Age at tibia scan (years)*	70.5 (5.3)
Height (cm)	160.5 (6.0)
Weight (kg)	68.8 (12.4)
BMI (kg/m^2^)	26.7 (4.8)
Total hip bone mineral density	0.84 (0.11)

	*N* (%)
Physically active compared to others	
Not at all	0 (0.0)
A little	39 (12.2)
Somewhat	164 (51.2)
Very	117 (36.6)
Current smoker	18 (5.6)
Alcoholic drinks per week	
None	64 (20.0)
1–6	133 (41.6)
7–13	95 (29.7)
14–20	22 (6.9)
>20	6 (1.9)
Education	
Below GCSE	78 (24.3)
CSE O level/GCSE	108 (33.6)
A level	35 (10.9)
Degree	100 (31.2)
Use of anti-osteoporotic medication	37 (12.0)
Ever used oestrogen/hormone replacement therapy	160 (50.6)
Years since last menstrual period	
<10	100 (32.1)
10–19	130 (41.7)
20–29	65 (20.8)
>29	17 (5.4)

* *n* = 306 tibia, 321 radius

Participants were asked how physically active they were compared to other women of the same age

### HRpQCT Parameters and Fracture Status

The associations between fracture history and individual radius and tibia HRpQCT parameters are presented in Table 2. History of fracture was associated with lower radial cortical porosity (*p* = 0.012), trabecular density (*p* = 0.001) and trabecular number (*p* < 0.001), and higher trabecular separation (*p* < 0.001). These associations were robust to adjustment. At the tibia, history of fracture was associated with lower trabecular density (*p* = 0.002) and number (*p* < 0.001), and higher trabecular separation (*p* < 0.001); associations regarding trabecular number and trabecular separation were robust to adjustment.

**Table 2:** Standard deviation difference in mean HRpQCT parameters (95% CI) for individuals who experienced a fracture since age 45 compared to those who did not

HRpQCT parameter	Unadjusted		Adjusted*	
	Estimate (95% CI)	*p* value	Estimate (95% CI)	*p* value
Radius				
Total area	0.12 (−0.16,0.39)	0.412	0.02 (−0.24,0.28)	0.869
Trabecular area	0.14 (−0.14,0.42)	0.317	0.03 (−0.23,0.29)	0.848
Cortical area	−0.11 (−0.38,0.17)	0.451	0.02 (−0.25,0.29)	0.890
Cortical thickness	−0.19 (−0.47,0.08)	0.170	−0.05 (−0.32,0.22)	0.710
Cortical volumetric density	−0.04 (−0.32,0.23)	0.752	0.08 (−0.19,0.34)	0.581
Cortical porosity	−0.35 (−0.63,−0.08)	0.012	−0.31 (−0.60,−0.03)	0.033
Trabecular volumetric density	−0.45 (−0.73,−0.18)	0.001	−0.35 (−0.63,−0.07)	0.016
Trabecular number	−0.66 (−0.92,−0.39)	<0.001	−0.59 (−0.86,−0.32)	<0.001
Trabecular thickness	0.08 (−0.20,0.35)	0.587	0.17 (−0.13,0.47)	0.270
Trabecular separation	0.59 (0.32,0.86)	<0.001	0.50 (0.23,0.78)	<0.001
Tibia				
Total area	0.25 (−0.03,0.53)	0.078	0.09 (−0.13,0.32)	0.417
Trabecular area	0.27 (−0.01,0.55)	0.062	0.10 (−0.13,0.33)	0.380
Cortical area	−0.15 (−0.43,0.13)	0.284	−0.03 (−0.29,0.23)	0.809
Cortical thickness	−0.24 (−0.52,0.04)	0.089	−0.13 (−0.39,0.14)	0.354
Cortical volumetric density	−0.18 (−0.46,0.10)	0.203	−0.05 (−0.31,0.21)	0.720
Cortical porosity	−0.03 (−0.31,0.25)	0.844	−0.11 (−0.39,0.18)	0.458
Trabecular volumetric density	−0.43 (−0.71,−0.16)	0.002	−0.28 (−0.57,0.01)	0.060
Trabecular number	−0.49 (−0.77,−0.22)	<0.001	−0.42 (−0.69,−0.15)	0.003
Trabecular thickness	−0.03 (−0.31,0.25)	0.838	0.11 (−0.19,0.41)	0.463
Trabecular separation	0.50 (0.22,0.78)	<0.001	0.40 (0.13,0.67)	0.004

* Adjusted for age at time of HRpQCT scan, height, BMI, physical activity, smoking status, alcohol consumption, education, time since last period, use of anti-osteoporotic medication, and use of oestrogen/hormone replacement therapy

*vBMD* volumetric bone mineral density

### Cluster Analysis of Radial HRpQCT Parameters

**Fig. 1:**
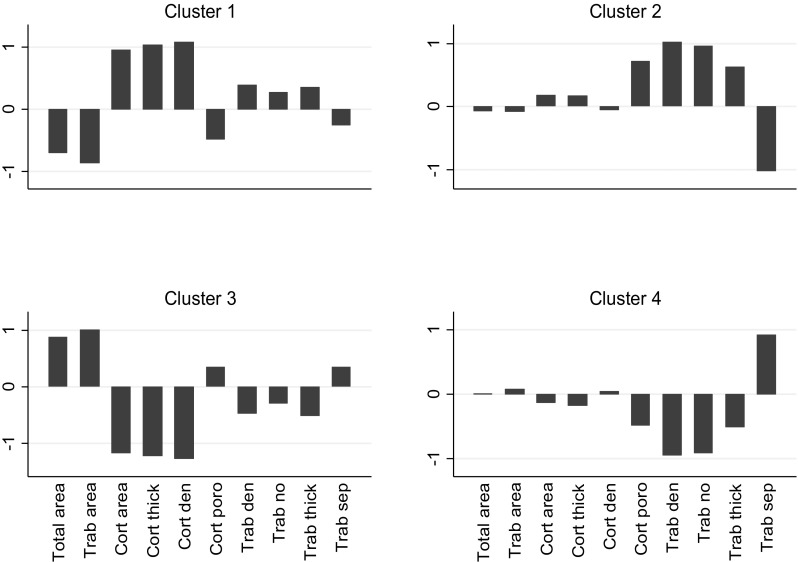
Means of standardized radial HRpQCT parameters according to each cluster. HRpQCT radial parameters included the following: total area, trabecular area, cortical area, cortical thickness, cortical density, cortical volumetric density, cortical porosity, trabecular volumetric density, trabecular number, trabecular thickness and trabecular separation

Four clusters were obtained. The summary statistics of the standardized HRpQCT parameters, hip aBMD and fracture prevalence according to the different clusters are illustrated in Table 3 and Fig. 1.

**Table 3:** Mean (SD) parameters by cluster analysis group (4 clusters of radial HRpQCT parameters obtained)

Parameter	Cluster 1 (*n* = 84)	Cluster 2 (*n* = 80)	Cluster 3 (*n* = 71)	Cluster 4 (*n* = 86)
HRpQCT (standardized)				
Total area	−0.69 (0.84)	−0.07 (0.75)	0.89 (0.86)	0.01 (0.90)
Trabecular area	−0.86 (0.79)	−0.09 (0.68)	**1.01 (0.76)**	0.08 (0.80)
Cortical area	0.97 (0.69)	0.17 (0.70)	−**1.18 (0.65)**	−0.13 (0.60)
Cortical thickness	**1.04 (0.68)**	0.17 (0.63)	−**1.22 (0.59)**	−0.17 (0.54)
Cortical volumetric density	**1.08 (0.58)**	−0.05 (0.60)	−**1.27 (0.55)**	0.04 (0.60)
Cortical porosity	−0.49 (0.75)	0.71 (0.78)	0.35 (1.00)	−0.48 (0.88)
Trabecular volumetric density	0.39 (0.62)	**1.02 (0.63)**	−0.47 (0.66)	−0.95 (0.65)
Trabecular number	0.27 (0.66)	0.96 (0.68)	−0.29 (0.79)	−0.92 (0.74)
Trabecular thickness	0.36 (0.85)	0.63 (0.73)	−0.51 (0.84)	−0.52 (1.00)
Trabecular separation	−0.26 (0.65)	−**1.03 (0.75)**	0.35 (0.71)	0.92 (0.63)
DXA				
Total hip aBMD	0.89 (0.11)	0.89 (0.10)	0.78 (0.09)	0.78 (0.10)
p value	reference	0.943	<0.001	<0.001
Fracture history				
Any fracture^a^	11 (13.1%)	13 (16.3%)	11 (15.5%)	28 (32.6%)
RR (95% CI) of fracture	1.00 (*reference*)	1.24 (0.59, 2.61)	1.18 (0.55, 2.57)	2.49 (1.32, 4.67)
p value	reference	0.569	0.671	0.005

*p* values calculated using a Poisson regression model with a robust variance estimator. *p* values for differences in hip aBMD were calculated using linear regression. *p* values are for differences compared to Cluster 1 (lowest risk)

Bold if mean >1 SD from sample mean

*RR* relative risk, *CI* confidence interval

^a^ *N* (%)

In Cluster 4, there was a trend towards lower trabecular density and number and higher trabecular separation compared to the analysis sample (differences in means >0.9 SDs). In this cluster, hip aBMD was significantly lower (*p* < 0.001) and individuals had a significantly higher risk of fracture [relative risk (95% CI) compared to Cluster 1: 2.49 (1.32, 4.67), *p* = 0.005]. In contrast to a trabecular deficiency pattern in Cluster 4, Cluster 3 showed differences predominantly in cortical parameters with trend towards lower cortical area, cortical thickness and cortical density, and higher trabecular area compared to the measured sample (differences in means exceeded one SD). Hip aBMD in this cluster was significantly lower, but there was no significant difference in fracture risk compared to Cluster 1.

Similarly in Cluster 1, differences were predominantly in cortical parameters, but here with a trend towards higher cortical area, cortical thickness and cortical density compared to the measured sample (differences in means >0.95 SDs). As expected, total hip aBMD was the highest and fracture risk was the lowest in this cluster.

Cluster 2 had higher trabecular density and lower trabecular separation, but there were no other HRpQCT parameter with means that differed by more than one SD compared to the sample mean. There was no significant difference in hip aBMD or fracture risk in this cluster. Adjustment for hip aBMD throughout did not remove previously observed associations, except that the associations for trabecular density of the radius were attenuated when additionally adjusted for aBMD.

### Cluster Analysis of Tibial HRpQCT Parameters

Four clusters were obtained among the 306 participants with complete data for the tibia parameters. The summary statistics of the standardized HRpQCT parameters, hip aBMD and fracture prevalence according to the different clusters are illustrated in Table 4.

**Table 4:** Mean (SD) parameters by cluster analysis group (4 clusters of tibial HRpQCT parameters obtained)

Parameter	Cluster 1 (*n* = 83)	Cluster 2 (*n* = 63)	Cluster 3 (*n* = 77)	Cluster 4 (*n* = 83)
HRpQCT (standardized)				
Total area	−0.62 (0.71)	−0.82 (0.77)	**1.01 (0.66)**	0.31 (0.64)
Trabecular area	−0.56 (0.67)	−**1.00 (0.71)**	**1.04 (0.60)**	0.35 (0.58)
Cortical area	0.05 (0.60)	**1.20 (0.70)**	−0.67 (0.87)	−0.34 (0.80)
Cortical thickness	0.11 (0.71)	**1.29 (0.66)**	−0.78 (0.73)	−0.36 (0.63)
Cortical volumetric density	0.23 (0.58)	**1.19 (0.65)**	−0.67 (0.82)	−0.51 (0.78)
Cortical porosity	0.00 (0.94)	−0.77 (0.84)	0.01 (0.85)	0.59 (0.91)
Trabecular volumetric density	−0.28 (0.71)	0.60 (0.78)	−**1.03 (0.65)**	0.78 (0.63)
Trabecular number	−0.75 (0.68)	0.69 (0.61)	−0.52 (0.88)	0.71 (0.74)
Trabecular thickness	0.50 (0.88)	0.05 (0.85)	−0.89 (0.79)	0.28 (0.85)
Trabecular separation	0.72 (0.64)	−0.71 (0.65)	0.63 (0.80)	−0.76 (0.72)
DXA				
Total hip aBMD	0.79 (0.09)	0.94 (0.1)	0.78 (0.1)	0.86 (0.09)
p value	<0.001	reference	<0.001	<0.001
Fracture history				
Any fracture^a^	19 (22.9%)	7 (11.1%)	24 (31.2%)	11 (13.3%)
RR (95% CI) of fracture	2.06 (0.92, 4.60)	1.00 (*reference*)	2.81 (1.29, 6.09)	1.19 (0.49, 2.91)
p value	0.078	reference	0.009	0.698

*p* values calculated using a Poisson regression model with a robust variance estimator. *p* values for differences in hip aBMD were calculated using linear regression. *p* values are for differences compared to Cluster 1 (lowest risk)

Bold if mean >1 SD from sample mean

*RR* relative risk, *CI* confidence interval

^a^ *N* (%)

Fracture risk was lowest and hip aBMD was highest in Cluster 2. This cluster had lower trabecular area and higher cortical area, thickness and density compared to the analysis sample (differences in means exceeded one SD). Cluster 3 had the highest risk of fracture and the lowest hip aBMD; this cluster was characterised by higher total and trabecular area and lower trabecular density compared to the analysis sample. For the other clusters, none of the tibia parameters differed from the analysis sample by more than one SD.

## Discussion

This study demonstrated that microstructural parameters of the bone evaluated by HRpQCT are different between healthy participants and fracture participants at skeletal regions containing predominantly trabecular bone. Trabecular parameters assessed by HRpQCT provided additional skeletal information to that captured from the standard areal bone mineral density (BMD) measurements by DXA. A cluster analysis of the radial and tibial HRpQCT parameters derived one cluster with a significantly higher fracture risk. Individuals in this cluster had lower trabecular density and number, and consequently higher trabecular separation compared to the wider sample. In this cluster, hip aBMD was significantly lower.

An aim of this study was to attempt to replicate findings from the Hertfordshire Cohort Study [12]. We showed that various indices of bone microarchitecture of the radius, most notably cortical porosity, trabecular density, trabecular number and trabecular separation, appeared to be compromised among postmenopausal UK women with a previous history of fracture. These results are in agreement with findings from Hertfordshire [12] and another published study [11] suggesting that alterations of trabecular architecture are likely to play an important role in skeletal fragility associated with osteoporosis [21]. In this study, the results for trabecular parameters described above remained robust to adjustments for demographic and lifestyle factors indicating that results are not due to confounding. Interestingly, and perhaps unexpectedly, history of fracture was associated with lower cortical porosity. Fracture cases had higher cortical area, consistent with findings from other cohorts, however, they also had higher cortical vBMD which is probably due to the lower porosity. This observation has now been made in both the Hertfordshire and GLOW cohorts, and warrants further investigation.

We did see differences in relationships at the radius and tibia which require validation in other samples. This may reflect technical differences in acquisition at the two sites, or differences due to the weight bearing/non-weight bearing nature of the two sites. Fractures in this group were more typically reported at the distal radius, which may also be relevant.

Cluster analysis of the radial HRpQCT parameters demonstrated one phenotype associated with higher risks of fracture. The altered parameters in this cluster included lower trabecular density and number and higher trabecular separation. This is consistent with the previous study on cluster analysis of bone microarchitecture from HRpQCT and fracture risk [12]. Similarly hip aBMD was low in this cluster when compared to the reference cluster in both studies. Interestingly, there was one more very similar phenotype derived by cluster analysis in both studies. It was characterised by higher trabecular area and lower cortical area, thickness and density. In this study, this cluster was not associated with higher fracture risk which is in contrast to the previously published study, where the participants were recruited from the Hertfordshire Cohort Study (HCS). Participants of the HCS were older, of mean age with and without a fracture 77.2 (2.4) and 76.0 (2.6), respectively, compared to participants in our study [mean age of 70.6 (5.4) at time of scan]. In the HCS, there were also differences in phenotype between genders where one cluster associated with high rates of fracture was characterised by low cortical thickness and density in men and women, but in men only, a cluster characterised by higher total and trabecular area was associated with increased fracture risk. Moreover, this cluster in men was not associated with low femoral neck areal BMD. In GLOW, only females were recruited but higher trabecular and total area (in addition to lower trabecular density) were the characteristics of Cluster 3 significantly associated with fracture risk, suggesting a consistency of phenotype.

This study has some limitations. As it is a cross-sectional study, causality cannot be determined since it is not possible to know whether bone microarchitecture changes preceded the fracture. Well-designed prospective studies providing longitudinal data are therefore very important. Although it is reported that cluster analysis models can be very unstable, which could affect the generalizability of the findings in this study, the results were largely consistent to a study by Edwards et al. [12].

In conclusion, this study indicates a phenotype with a significantly higher fracture risk, using cluster analysis of radial and tibial HRpQCT parameters. This approach may have clinical utility in patients where such scans are available, as it allows the incorporation of a large number of variables acquired during a scan to be combined into a bone phenotype that may be more useful for a clinician and patient alike. While our observations were generally in accord with those found in the Hertfordshire Cohort Study, we did note some differences that may reflect the demographic differences between the two groups, particularly age. Given the number of cohorts where HRpQCT data are available, we would welcome attempts at similar analyses. Ultimately our study adds to the growing body of evidence demonstrating distinct phenotypes of bone fragility, which may have implications for targeted prevention and treatment of osteoporosis in the future. Further research is required to examine the identified phenotype and its ability to predict future fracture.
